# Lafutidine 10 mg versus Rabeprazole 20 mg in the Treatment of Patients with Heartburn-Dominant Uninvestigated Dyspepsia: A Randomized, Multicentric Trial

**DOI:** 10.1155/2011/640685

**Published:** 2011-05-05

**Authors:** Bhupesh Dewan, Nisha Philipose

**Affiliations:** ^1^Medical Services Department, Zuventus Healthcare Ltd, 5119 “D” Wing, Oberoi Garden Estate, Chandivilli, Mumbai 400072, India; ^2^Clinical Research Associate, Medical Services Department, Zuventus Healthcare Ltd, Mumbai 400072, India

## Abstract

*Background*. Empirical therapy with antisecretory agents like PPIs and H2RAs has long been the traditional approach in the initial management of uninvestigated dyspepsia. *Aim*. The objective of the study was to examine relief of dyspepsia with lafutidine, a second-generation H_2_-RA, and rabeprazole and to compare their efficacy. *Methods*. This was a randomized, open, comparative trial in adult uninvestigated dyspeptic patients, who had at least moderate severity of symptoms, defined as a score of ≥4 on a 7-point global overall symptom (GOS) scale, and were randomized to receive once daily either lafutidine 10 mg or rabeprazole 20 mg for 4 weeks. *Results*. A total of 236 patients were enrolled, out of which 194 patients were included in the analysis. At the end of week 4, a significant difference was observed for symptom relief (lafutidine 89.90% versus rabeprazole 65.26%, *P* < .01) and symptom resolution (lafutidine 70.71% versus rabeprazole 25.26%, *P* < .01). Both the drugs were well tolerated. *Conclusion*. Both lafutidine and rabeprazole provide symptom relief in patients with heartburn-dominant uninvestigated dyspepsia. The present study confirms the appropriateness of lafutidine as an empiric treatment and superior efficacy for primary care practice patients with dyspepsia.

## 1. Introduction

Dyspepsia is believed to be common worldwide [1]. In the Indian population, dyspepsia was found to be more common in subjects aged 40 years or younger [2]. It is more prevalent in the metros, where it is reported by almost one-third of the population [3]. 

The Canadian Dyspepsia Working Group definition states that *“Dyspepsia is a symptom complex of epigastric pain or discomfort thought to originate in the upper gastrointestinal tract, and it may include any of the following symptoms: heartburn, acid regurgitation, excessive burping/belching, increased abdominal bloating, nausea, feeling of abnormal or slow digestion, or early satiety*” [4]. Patients with dyspeptic symptoms who are not candidates for early endoscopy are termed “uninvestigated dyspeptics” [5]. 

The American College of Gastroenterology Guidelines, and the NICE guidelines from UK, for the management of dyspepsia have among others stated two commonly used strategies, namely test and treat strategy and acid suppression therapy [6, 7]. For those in whom the predominant symptoms are thought to be acid related, such as epigastric pain or burning, a trial of acid suppression is often recommended if the patient is under 45–55 years of age without obvious organic disease [8]. 

Empirical therapy with antacids, antisecretory, and prokinetic agents has long been the traditional approach for most primary care physicians in the initial management of patients with uninvestigated dyspepsia [9]. Since their introduction in the mid-1970s, H_2_-RA have gained wide acceptance as antisecretory drugs with proven efficacy in the treatment of peptic ulcers and gastro-oesophageal reflux disease. There are currently four drugs in this category in use: cimetidine, ranitidine, famotidine, and nizatidine [10]. 

Lafutidine was developed as a second-generation H_2_-RA with an increased action on the gastric mucosal defensive capacity. It has been reported that the gastroprotective effect of lafutidine is independent of its acid antisecretory activity [11]. In addition to being a potent H2 receptors antagonist [12], lafutidine also activates capsaicin-sensitive afferent neurons and stimulates the release of calcitonin gene-related peptide (CGRP), which inhibits acid secretion and stimulates mucosal blood flow [13–15]. The gastro protective action of lafutidine includes increase in mucin biosynthesis via stimulation of nitric oxide production [11, 16], increasing the thickness of the surface mucus gel layer [17], and maintaining gastric mucosal blood flow and bicarbonate response [18]. Lafutidine has been elaborately studied internationally in various indications like gastritis, and gastric and peptic ulcer, while recent focus has also shifted to its use in gastroesophageal reflux disorder (GERD) [19–23]. 

The objective of the present study was to compare the efficacy of lafutidine and rabeprazole in the management of heartburn-dominant uninvestigated dyspepsia in Indian primary care practice.

## 2. Methods

The current study (study no.: Emc/GASTR/LAFUT/12/2008) was a prospective, multicentric study, carried out at 5 centers in India. The study was conducted in accordance with the Declaration of Helsinki and its subsequent amendments and in compliance with the ICH-GCP (International Conference on Harmonization-Good Clinical Practice) Guidelines. All patients were required to give their written informed consent before entering into the study. The protocol was approved by “Jagruti-Independent Ethics Committee”, Mumbai, India, for each investigational centre in August 2009.

### 2.1. Patients

Patients aged 18 years or older were eligible if they had a minimum of 1-month history of dyspepsia (including symptoms of heartburn and/or epigastric pain and/or bloating) with at least one moderate-to-severe symptom (score ≥4 on a 7-point global overall symptom (GOS) scale) on at least three of the 7 days before randomization. The main exclusion criteria were presence of alarm features (unintentional weight loss, persistent vomiting, dysphagia, haematemesis, melaena, fever, jaundice, or anaemia), irritable bowel syndrome or serious concomitant disease, previous history of gastrointestinal disease (including peptic ulcers, malignancy, oesophageal dysmotility, a previous endoscopic or radiological diagnosis of GERD, and Barrett's oesophagus), recent gastrointestinal surgery, that is, within 30 days (except appendectomy, colonic resection, and cholecystectomy), treatment with nonsteroidal anti-inflammatory drugs (NSAIDs), acetylsalicylic acid (>325 mg/day), H_2_-RAs, PPIs, prokinetic agents, misoprostol, or sucralfate 15 days prior to enrollment, and a known history of hypersensitivity to the study medications.

Patients who had previously been included or who had participated in any other clinical trial within one month before enrollment into the current study were excluded. Women who were pregnant, lactating, or who desire to become pregnant during the study were not enrolled. Patients who were drug or alcohol abusers or suffering from any other condition associated with poor compliance were excluded from the study.

### 2.2. Study Design

The current study was a randomized, comparative-controlled clinical trial, wherein a target sample size of at least 200 patients with clinical signs and symptoms of dyspepsia were to be enrolled. Each investigator of the five participating centers was to enroll 50 patients. Based on the inclusion/exclusion criteria as stated in the protocol, the participant was considered eligible for participating in the study by the investigator. Once enrolled, the patients received treatment for a period of four weeks (28 days) with either lafutidine or rabeprazole, as per the two-treatment computer-generated randomization sequence, using WINPEPI Version 8.6. Each patient was supplied with the medication (lafutidine or rabeprazole) which would suffice for the treatment duration of 4 weeks. The patient's medication supply was divided into two parts, administered at the time of enrollment and on day 14 (after week 2). Thus, each investigator was provided with medication supply for 50 patients: two sealed, tamperproof, labeled, plastic containers containing 14 tablets of lafutidine or rabeprazole for each patient. During this study period, the patients were assessed for the clinical signs and symptoms and the response to the treatment after week 2 (day 14) and week 4 (day 28). Laboratory assessment was performed at the time of enrollment and at the end of the study.

### 2.3. Treatment

Patients received the study medications as per the randomization chart for duration of 28 days. The dosage regimen of the investigational drug was one lafutidine 10 mg tablet (manufactured by: Emcure Pharmaceuticals Ltd; Batch no: FD/388/09) to be taken once daily for 28 days, while that of the control group was one rabeprazole 20 mg tablet (manufactured by: Windlas Biotech Limited; Batch no: ZYB09003) to be taken once daily for 28 days. Adherence to the compliance for medications was calculated using pill count of returned medications after two and four weeks of treatment.

### 2.4. Assessments

Patients were asked to indicate the overall severity of their dyspepsia symptoms during the previous two days using the validated 7-point GOS ccale: (1) no problem, (2) minimal problem (can be easily ignored without effort), (3) mild problem (can be ignored with effort), (4) moderate problem (cannot be ignored but does not influence my daily activities), (5) moderately severe problem (cannot be ignored and occasionally limits my daily activities), (6) severe problem (cannot be ignored and often limits my concentration on daily activities), and (7) very severe problem (cannot be ignored, markedly limits my daily activities and often requires rest). 

A patient reporting at least moderate severity (score ≥4) for an individual symptom was considered to be symptomatic for that specific symptom. Reassessment was performed at the end of second week (day 14) and fourth week (day 28).

The *“overall treatment evaluation”* (OTE) assessed the patient's perspective on symptom relief on a 6-point Likert scale ((1) the treatment made me a lot worse; (2) the treatment made me slightly worse; (3) the treatment made no change to my symptoms; (4) the treatment made me slightly better; (5) the treatment made me a lot better; (6) the treatment completely relieved my symptoms). The “*overall patient satisfaction survey”* (OPSS) evaluated patient satisfaction with treatment using a 6-point Likert scale ranging from completely satisfied (1) to completely dissatisfied (6). Finally, the frequency and severity of adverse events were evaluated at each visit while clinically important changes in the laboratory data during the study were assessed.

### 2.5. Study Outcome

The primary study outcome was determination of the “*symptom relief*,*” *that is, the proportion of the patients achieving a score of GOS ≤ 2 for epigastric pain, heartburn, and abdominal bloating after two and four weeks of treatment.

The secondary outcomes included the determination of the resolution of the symptoms of dyspepsia, that is, the proportion of the patients achieving* “symptom resolution*” (GOS = 1) and *“symptom improvement”* (ΔGOS ≥ 2) for epigastric pain, heartburn, and abdominal bloating after 2 and 4 weeks of treatment.

The proportions of the patients for the analysis of patient's perspective on symptom relief with a score of ≥4 on the 6-point Likert scale at the end of week 2 and week 4 were evaluated. The proportion of the patients with a score of ≤3 on the 6-point Likert scale for overall patient satisfaction survey (OPSS) was also assessed and compared at the end of the study.

### 2.6. Statistical Analysis

The change in severity of individual symptoms between the visits in each treatment group was compared by Wilcoxon rank sum test. A comparative evaluation for the mean score reduction between the two groups was performed by Mann-Whitney *U*-test. The proportions of patients with symptom relief, resolution, and improvement over the 4-week treatment period were reported as “percentage” along with their “95% confidence interval” (CI), and the comparison between the treatment groups was performed using Fisher's exact test. The treatment assessment results reported by the patients were summarized in frequency tables by treatment groups. The comparison of the mean scores of treatment assessment results was performed by Mann-Whitney *U*-test. All data are presented as mean ± standard deviation (SD) unless stated otherwise. *P* value less than  .05 was considered significant.

## 3. Results

### 3.1. Patient Characteristics and Disposition

**Figure 1:**
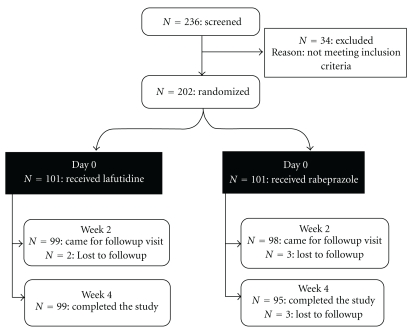
CONSORT flow diagram showing the number of patients enrolled in the study randomized to each treatment group and reasons for discontinuation.

Of the 236 patients cumulatively enrolled by the 5 centres, 194 patients completed the study, of which 99 patients received lafutidine while 95 of them received rabeprazole.  Figure 1 shows the consolidated standards of reporting trials (CONSORT) flow chart of patients throughout the study. Thus, the study population comprised 194 patients who were evaluated for resolution of clinical symptoms of dyspepsia and for the incidence of adverse events. Patients were enrolled over a 4-month period, from August 2009 to November 2009. The study was completed by first week of December 2009. Baseline demographic data are shown in Table 1. It was observed that obesity was uncommon in the group of patients enrolled in this study.

In the study population, most of the patients suffered from one or more symptoms. The proportion of patients with moderate to severe dyspepsia symptoms (score ≥4 on a 7-point global overall symptom (GOS) scale) is reported in Table 1. The overall prevalence of all the 3 symptoms with a score ≥4 on GOS scale was found in 81 (81.81%) and 70 (73.68%) of the patients in lafutidine and rabeprazole group, respectively. 

Adherence to therapy over the 4-week duration was excellent as 100% and 98.94% of the patients took the medication as per the dosage regimen in the lafutidine and rabeprazole groups, respectively. None of the patients missed the dose for more than two successive days anytime during the study period.

### 3.2. Overall Symptom Assessment

The proportion of patients achieving symptom relief, symptom resolution, and symptom improvement for the overall severity of their dyspepsia symptoms in each treatment group is tabulated in Table 2. At the end of week 2, the proportion of patients achieving symptom relief (GOS ≤ 2) and symptom resolution (GOS = 1) between the two treatment groups was not significant. By the end of week 4, the proportion of patients with symptom relief and symptom resolution was significantly higher in patients in lafutidine group than rabeprazole group. In terms of symptom improvement (ΔGOS ≥ 2), lafutidine and rabeprazole groups were not significantly different (Table 2). 

### 3.3. Response by Individual Symptom

**Figure 2:**
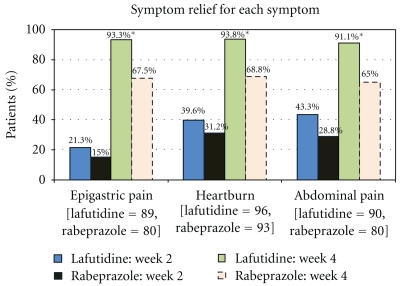
Proportion of patients who reported symptom relief for each individual symptom from baseline. The number of patients listed for each symptom is the number of patients that reported that symptom with a GOS score ≥4 at baseline.*P < .01* between the two treatment groups at week 4. *

**Figure 3:**
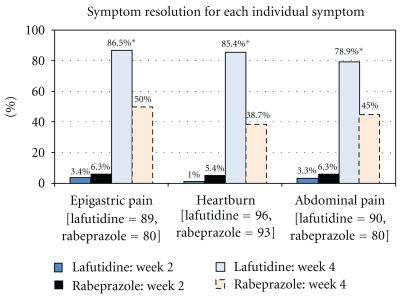
Proportion of patients who reported symptom resolution for each individual symptom from baseline. The number of patients listed for each symptom is the number of patients that reported that symptom with a GOS score ≥4 at baseline.*P < .01* between the two treatment groups at week 4. *

A patient was considered to be symptomatic for an individual symptom if that symptom was reported to be at least moderate (score ≥4) in severity at baseline. Patients in the lafutidine group reported a better improvement in epigastric pain, heartburn, as well as abdominal bloating as compared to rabeprazole group, which was statistically significant, after 4 weeks of treatment (Figures 2 and 3).

At baseline, the mean score for each symptom did not differ significantly between the two treatment groups. A significant reduction in the mean score for each symptom from the mean baseline score at week 2 and week 4 was observed in both lafutidine as well as rabeprazole group (Table 3).

### 3.4. Patient's Assessment of the Treatment

In the *“overall treatment evaluation”* (OTE) assessing the patient's perspective on symptom relief, lafutidine was better than rabeprazole. At the end of week 4, among the total patients in each group, a score of 4 and above (slightly better to completely relieved my symptoms) was reported by 95.96% and 87.37% in the lafutidine and rabeprazole group, respectively. At the end of the study, 74.75% of the lafutidine-treated patients stated that the treatment had completely relieved their symptoms, compared to 32.63% of the rabeprazole-treated patients (Table 4). The mean score of patient's perspective on symptom relief was significantly higher in lafutidine group (5.52 ± 0.95 versus 4.83 ± 1.05 in rabeprazole group, *P* < .0001).

**Figure 4:**
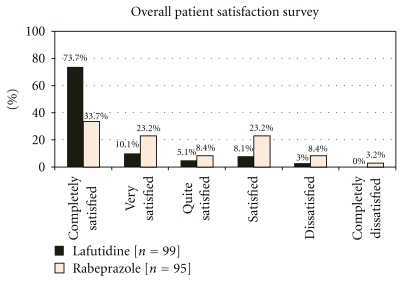
Frequency distribution of satisfaction survey responses at the end of the treatment phase.

At the 4-week assessment, when patients were asked to rate the overall satisfaction of their current treatment, 88.89% and 65.26% in the lafutidine and rabeprazole group respectively reported a score of 3 or less *(quite satisfied to very satisfied).* The satisfaction survey response reported by the patients is shown in Figure 4. The mean score of overall patient satisfaction was significantly higher in lafutidine group at the end of the study (1.57 ± 1.1 versus 2.59 ± 1.51 in Rabeprazole group, *P* < .0001).

### 3.5. Adverse Events

Both the treatment groups were found to be safe and well tolerated. The frequency of adverse events reported was similar in both treatment arms although the type of adverse event reported in each treatment group was different. None of the adverse events experienced by the subjects during this study were judged as serious. In lafutidine group, one patient reported constipation which was rated “*moderate*” in severity at the end of week 2 and 4 visit, while another patient reported it only at the end of week 4 which was rated as “*mild*” in severity. Diarrhea was reported by 1 patient by week 4 in the lafutidine group which was rated as “*mild*” in severity. In case of rabeprazole group, 3 patients reported headache as an adverse event at the end of week 2 which was rated as “*mild*” in severity. Thus, the overall incidence of adverse events reported were headache (2.65%) in rabeprazole group and constipation (1.73%) and diarrhoea (0.86%) in lafutidine group. There were no clinically relevant laboratory abnormalities in either treatment group.

## 4. Discussion

Dyspepsia is a common problem encountered in general practice. There have been many dyspepsia management strategies and guidelines published in recent years. The British Society of Gastroenterology (BSG) Dyspepsia Management Guidelines state that it is acceptable to initiate a single course of treatment with an antisecretory agent, for 2 to 4 weeks, in patients under the age of 45 years who are experiencing troublesome dyspepsia but exhibit no alarm symptoms. Endoscopy is not recommended in this group of patients without evidence of the presence of Helicobacter pylori together with persistent symptoms. Treatment without investigation is, therefore, appropriate in many cases [24].

The present study focuses on uninvestigated dyspeptics, that is, patients with dyspepsia symptoms who are not candidates for early endoscopy. Endoscopy was not performed at enrolment in the present study; although it would have allowed risk stratification with respect to oesophagitis severity and H. pylori infection, it would also have introduced the potential for bias in a study intended to assess symptom response to empiric therapy in a primary care environment.

The prevalence of uninvestigated dyspepsia in India has been reported to be 30.4% [25], while in another study, the ethnic prevalence of esophagitis was 8.5% among Indians [26]. Helicobacter pylori infection is common in the Indian subcontinent. Exposure occurs in childhood and approximately 80% of adults have been infected at some time [27].

The GOS scale used in this study is a validated outcome measure and has been used successfully in the CADET (Canadian Adult Dyspepsia Empiric Treatment) Clinical Trials Program. In the validation of the GOS study, it has been shown that a change in GOS from ≥4 to ≤2 is rated as clinically important by patients [28].

In this study, both rabeprazole and lafutidine showed positive effects on the symptoms of epigastric pain, heartburn, and abdominal bloating. A statistically nonsignificant difference in response was observed for both the treatments at the end of second week; however, at week 4, the differences were statistically significant. No serious or life-threatening adverse events were observed in either treatment groups and the absence of any significant change in the laboratory parameters confirms the safety of both the drugs.

Meta-analysis evaluating the efficacy of H_2_-RA in functional (nonulcer) dyspepsia has shown that H_2_-RAs are superior to placebo [12]. Proton pump inhibitors (PPIs) are more potent inhibitors of gastric acid secretion than the first generation H_2_-RAs [29]. Most clinicians acknowledge the advantage of PPIs over H_2_-RAs, because the former exert stronger and longer acid suppression than the latter [30]. Clinical trials comparing a PPI (omeprazole and lansoprazole) and H_2_-RA (ranitidine and cimetidine) demonstrates superiority of PPI over H_2_-RA (ranitidine) in dyspepsia [10, 24, 31]. 

There are only a few studies currently available evaluating the treatment for uninvestigated dyspepsia in comparisons to those for the treatment of functional dyspepsia or GERD. Empirical treatment of dyspepsia with PPI leads to an acceptable symptom relief in 54%–65% of the patients compared with 33%–53% with placebo [32]. Armstrong et al. reported resolution of only heartburn in 36.2% of the patients treated with omeprazole 20 mg a day [31]. In another study, an overall resolution of all the dyspepsia symptoms was observed 22.9% of the patients treated with esomeprazole 40 mg once daily [28]. In the current study, symptom resolution for all the dyspepsia symptoms was observed in 25.26% rabeprazole-treated patients, while in lafutidine-treated patients, this proportion was significantly higher, 70.71%. The better efficacy of lafutidine seen in this study can be attributed to the novel mechanism of action of lafutidine which includes gastric mucosal protective action mediated by capsaicin-sensitive afferent neurons, in addition to a potent antisecretory effect.

In conclusion, the current study shows that both lafutidine and rabeprazole are effective in relieving the symptoms of dyspepsia. Therapy with lafutidine, a second-generation H_2_-RA, may be more useful than rabeprazole in relieving symptoms of heartburn-dominant uninvestigated dyspepsia.

## Figures and Tables

**Table 1:** Demographic summary.

	Lafutidine (*N* = 99)	Rabeprazole (*N* = 95)
Gender		
Males, *n* (%)	62 (62.63)	53 (53.54)
Females, *n* (%)	37 (37.37)	41 (41.41)

Age (years)		
Mean ± SD	39.24 ± 11.82	40.47 ± 12.13
Median	38	38
Range	18–66	20–74

Weight (Kg)		
Mean ± SD	59.48 ± 10.36	61.43 ± 9.76
Median	59	60.5
Range	38–85	40–90

Symptoms (score ≥4 at baseline)		
Epigastric pain, *n* (%)	89 (89.89)	80 (84.21)
Heartburn, *n* (%)	96 (96.96)	93 (97.89)
Abdominal pain, *n* (%)	90 (90.9)	80 (84.21)

**Table 2:** Proportion of patients achieving symptom relief (GOS ≤ 2), symptom resolution (GOS = 1), and symptom improvement (ΔGOS ≥ 2).

		Lafutidine (*N* = 99) *N* (%) (95% CI)	Rabeprazole (*N* = 95) *N* (%) (95% CI)	Difference (95% CI)	*P* value
Symptom relief (GOS ≤ 2)	Week 2	15 (15.15) (8.74 to 23.76)	13 (13.68) (7.49 to 22.26)	1.50% (−9.40 to 12.40)	.840
	Week 4	89 (89.90) (82.21 to 95.05)	62 (65.26) (54.80 to 74.74)	24.60% (12.30 to 36.90)	<.001
Symptom resolution (GOS = 1)	Week 2	1 (1.01) (0.03 to 5.50)	5 (5.26) (1.73 to 11.86)	−4.30% (−10.20 to 1.70)	.113
	Week 4	70 (70.71) (60.71 to 79.43)	24 (25.26) (16.91 to 35.22)	45.40% (31.90 to 59.00)	<.001
Symptom improvement (ΔGOS ≥ 2)	Week 2	65 (65.66) (55.44 to 74.91)	51 (53.68) (43.15 to 63.98)	12.00% (−2.80 to 26.70)	.107
	Week 4	91 (91.92) (84.70 to 96.45)	85 (89.47) (81.49 to 94.84)	2.40% (−6.80 to 11.70)	.626

**Table 3:** Symptom score (Mean ± SD) at baseline and after 2 and 4 weeks.

	Lafutidine	Rabeprazole	*P* value for difference between treatments
	Mean score ± S.D. (*N* = 99)	Mean score ± S.D. (*N* = 95)	
*Epigastric pain*			
Baseline	4.86 ± 1.19	4.87 ± 1.42	NS
Week 2	2.86 ± 0.71*	3.17 ± 0.96*	.002
Week 4	1.28 ± 0.74*	1.91 ± 1.07*	<.0001

*Heartburn*			
Baseline	5.07 ± 0.99	5.13 ± 0.91	NS
Week 2	2.71 ± 0.70*	2.98 ± 0.94*	.0224
Week 4	1.28 ± 0.71*	2.04 ± 1.03*	<.0001

*Abdominal bloating*			
Baseline	4.92 ± 1.08	4.97 ± 1.25	NS
Week 2	2.60 ± 0.74*	2.97 ± 1.06*	.0075
Week 4	1.36 ± 0.83*	1.95 ± 1.00*	<.0001

*P Value less than  .001 versus baseline, within the group.

NS: not significant (*P* > .05).

**Table 4:** Frequency distribution of “patient's perspective on symptom relief” survey responses.

	At the end of week 2		At the end of week 4	
Patient's perspective on symptom relief of the treatment	Lafutidine	Rabeprazole	Lafutidine	Rabeprazole
	(*n* = 99)	(*n* = 95)	(*n* = 99)	(*n* = 95)
	*n* (%)	*n* (%)	*n* (%)	*n* (%)
Made me a lot worse	0 (0.00)	0 (0.00)	1 (1.01)	0 (0.00)
Made me slightly worse	0 (0.00)	0 (0.00)	0 (0.00)	1 (1.05)
Made no change to my symptoms	4 (4.04)	13 (13.68)	3 (3.03)	11 (11.58)
Made me slightly better	30 (30.30)	45 (47.37)	13 (13.13)	22 (23.16)
Made me a lot better	62 (62.63)	33 (34.74)	8 (8.08)	30 (31.58)
Completely relieved my symptoms	3 (3.03)	4 (4.21)	74 (74.75)	31 (32.63)
